# Regulation of human *MAPT* gene expression

**DOI:** 10.1186/s13024-015-0025-8

**Published:** 2015-07-14

**Authors:** Marie-Laure Caillet-Boudin, Luc Buée, Nicolas Sergeant, Bruno Lefebvre

**Affiliations:** Univ. Lille, UMR-S 1172, Inserm, CHU, 59000 Lille, France

**Keywords:** Tau, Tauopathy, *MAPT*, Alzheimer’s disease, Repeat sequences, CpG islands, Tau haplotype, Tau promoter, Tau splicing

## Abstract

The number of known pathologies involving deregulated Tau expression/metabolism is increasing. Indeed, in addition to tauopathies, which comprise approximately 30 diseases characterized by neuronal aggregation of hyperphosphorylated Tau in brain neurons, this protein has also been associated with various other pathologies such as cancer, inclusion body myositis, and microdeletion/microduplication syndromes, suggesting its possible function in peripheral tissues. In addition to Tau aggregation, Tau deregulation can occur at the expression and/or splicing levels, as has been clearly demonstrated in some of these pathologies. Here, we aim to review current knowledge regarding the regulation of human *MAPT* gene expression at the DNA and RNA levels to provide a better understanding of its possible deregulation. Several aspects, including repeated motifs, CpG island/methylation, and haplotypes at the DNA level, as well as the key regions involved in mRNA expression and stability and the splicing patterns of different mRNA isoforms at the RNA level, will be discussed.

## Introduction

Tau proteins are expressed primarily in the brain and, more precisely, in neurons. These proteins were discovered in 1975 and identified as important mediators of cerebral microtubule polymerization and stabilization [1] (reviewed in [2, 3]). Since then, other roles for Tau proteins have been demonstrated. Tau is involved in axonal transport (reviewed in [4, 5]), synaptic plasticity/function (reviewed in [6, 7]) and nucleic acid protection [8, 9], depending on its cellular localization (cell body/axon, cytoplasmic membrane, or nucleus).

The functional importance of Tau is underscored by the involvement of Tau deregulation in neurodegenerative diseases. Aggregation of hyperphosphorylated Tau proteins in degenerating neurons, which leads to the formation of neurofibrillary tangles, occurs in a group of pathologies termed tauopathies (reviewed in [3]). The relationship between Tau proteins and pathophysiology is supported by the identification of autosomal dominant mutations in the Tau gene, *MAPT,* in various tauopathies, such as frontotemporal dementia with parkinsonism linked to chromosome 17 (FTDP-17) (reviewed in [10, 11]). Although certain tauopathies are clearly pure neurodegenerative diseases, such as Alzheimer’s disease (AD), progressive supranuclear palsy (PSP), corticobasal degeneration (CBD), and FTDP-17, some are associated with the development of other pathologies, such as arteriovenous malformation [12], brain tumors (such as ganglion cell tumors) [13, 14], viral infection (such as subacute sclerosing panencephalitis (SSPE) [15] and postencephalitic parkinsonism [16]), developmental abnormalities (verrucose dysplasia [17]), Down syndrome [18], myotonic dystrophy (DM) [19], parkinsonism-dementia of Guam [16, 20], traumatic brain injury [21, 22], and Huntington’s disease [23].

Tau isoforms are translated from alternatively spliced mRNA, and some or all of these isoforms aggregate, depending on the pathology (reviewed in [3]). Deregulated Tau expression and missplicing have been reported in several pathologies. The direct involvement of a splicing defect has been clearly demonstrated for FTDP-17 and DM types 1 and 2 (reviewed in [24]). Some patients with amyotrophic lateral sclerosis (ALS) or frontotemporal lobar dementia (FTLD) exhibit the nearly complete absence of Tau protein in the cortex despite normal Tau mRNA expression. These latter two pathologies, ALS and FTLD, are characterized by the presence of ubiquitin-positive aggregates composed of *TDP*-*43* (transactive response DNA binding protein 43 kDa) [25–27], as reviewed in [28]. Furthermore, *MAPT* is a major candidate involved in the mechanism of 17q21.31 microdeletion syndrome, a pathology characterized by the microdeletion of a small chromosomal region (from 650 to 1,608 kb) containing several genes, including *MAPT*. The symptoms of 17q21.31 microdeletion syndrome include mental retardation, hypotonia and characteristic facial features. Pathological phenotypes have also been associated with microduplications or microtriplications containing *MAPT* [29–32]. One of the single-nucleotide polymorphisms (SNPs) within the *MAPT* locus has been found to be associated with AD in patients without ApoE e4 [33]. The *MAPT* locus is an important genetic risk factor for Parkinson’s disease (PD) [33–35]. Taken together, these data demonstrate the complexity of Tau expression in the brains of healthy individuals and patients with the above-mentioned diseases.

More recently, several reports have suggested that Tau interferes with certain pathologies involving tissues other than the brain. For example, Tau aggregation has been reported in the muscles of patients suffering from inclusion body myositis (IBM), an inflammatory muscle disease [36, 37]. Furthermore, Tau expression may have a prognostic or predictive value in some cancers affecting various tissues, such as breast [38, 39], prostate [40, 41], ovary [42, 43], bladder [44], and stomach cancers [45]. Tau expression could be related to certain sub-types of cancer; for example, it is increased in hormone-dependent breast cancer [39, 46]. Such an increase in Tau expression may result in resistance to microtubule-targeting drugs [43, 47–56].

Despite the importance of the deregulation of Tau expression/metabolism in many pathologies, the regulation of the expression of the *MAPT* gene, which encodes Tau protein, has been the subject of few articles; more articles have focused on the function of Tau protein or its roles in various pathologies. The most commonly studied aspects include *MAPT* haplotypes and Tau RNA splicing because of their involvement in certain tauopathies (for examples, see reviews [57–61]). Research regarding the epigenetic regulation of Tau expression is increasing. However, some aspects of Tau gene expression, such as the possible existence of different promoters and the potential role of the repeated motifs found in all *MAPT* sequences, have rarely been studied, despite the availability of abundant data in bioinformatics databases. The aim of this review is to provide a global synthesis of knowledge regarding the regulation of human *MAPT* gene expression at the DNA and RNA levels by evaluating data from the literature and bioinformatics databases. Several aspects will be discussed, including repeated motifs, CpG island/methylation, and haplotypes at the DNA level as well as the key regions involved in mRNA expression and stability (i.e., the 5′ and 3′ untranslated regions [UTRs]) and the splicing patterns of different mRNA isoforms at the RNA level.

## DNA regulatory elements involved in Tau expression

The human *MAPT* gene is a long (134 kb) gene located on chromosome 17q21. Its sequence is well conserved among mammals, exhibiting 97 to 100 % homology with primates. This observation is quite surprising considering the specific human susceptibility to developing tauopathies.

### Repeated sequences

Numerous repeated sequences are spread throughout the human genome. These sequences can be classified into two groups: transposons and satellites.

#### Transposon-type repeats

Transposon-type repeats can be classified into the following four categories: short interspersed nuclear elements (SINEs), long interspersed nuclear elements (LINEs), DNA transposons, and transposable elements with long terminal repeats (LTRs). All 4 categories of this type of repeat are found in the *MAPT* gene*.* Generally, SINEs are the most numerous, and among them, *Alu* sequences are the most frequent. *Alu* elements are 300-nucleotide (nt) sequences that occupy over 10 % of the human genome. These sequences contribute to genomic instability by eliciting gene mutations, altered gene expression and transcript splicing or by generating a new gene segment [62]. Among the 83 *Alu* sequences identified in the *MAPT* gene using the TranspoGene database [63], 56 are on the sense strand and 27 are on the antisense strand. These sequences are located within intronic sequences, independent of the considered isoform. *Alu* sequences have also been localized to the *MAPT* promoter in a region upstream of the first exon (E0) [64, 65]. In addition, other types of repeated sequences have been identified in the *MAPT* gene, some of which are located in the promoter [64]. However, the impact of these sequences on promoter activity is not well understood.

#### Simple sequence repeats (SSRs)/microsatellites and minisatellites

SSRs are short repeated sequences that are abundant throughout eukaryote genomes, are localized to intergenic or genic regions, and constitute an important class of genetic markers. Depending on the length of the repeat, SSRs can also be called microsatellites (1 to 13 bases per repeat) or minisatellites (14 to 100 bases per repeat) [66]. The roles of these microsatellites/minisatellites are varied and are not all well understood. However, some effects of these motifs have begun to be reported. For example, dinucleotide motifs have been identified as part of a general enhancer feature (i.e., non-cell type-specific enhancers) [67]. Furthermore, SSRs affect DNA structure, gene expression and genomic stability and can lead to a toxic gain-of-function in an RNA or protein when the number of repeats increases, as has been observed in a growing number of neurodegenerative diseases. For example, DM and Huntington’s disease involve an RNA and protein, respectively, with a toxic gain of function (reviewed in [68, 69]).

**Fig. 1:**
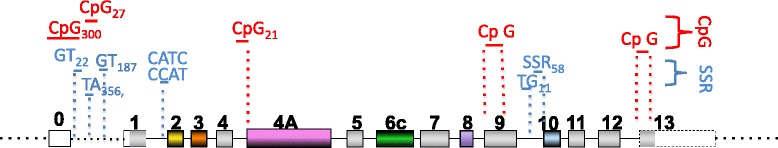
DNA satellite sequences and CpG islands in the *MAPT* gene. Only the repeats discussed in the text are indicated. The complete list can be found on the UCSC site. White, non-coding regions; color, coding regions; gray, constitutive coding exons. Yellow, orange, pink, green, purple and blue represent the alternative exons 2, 3, 4A, 6, 8 and 10, respectively

SSRs can be found throughout the *MAPT* gene using the Tandem Repeats Finder program (TRF/UCSC) [70]. Interestingly, the first genetic marker identified as over-represented in PSP patients was a dinucleotide repeat (11xTG, named TG_11_) downstream of exon 9 ([71, 72], reviewed in [60]). Some of these repeats are clearly distinguishable based on the following features: *(i)* their repeat length (4 sequences are longer than 20 nt, and the longest sequence is 58 nt); *(ii)* their localization throughout the gene (shorter sequences of less than 20 nt are located before exon E4, whereas the longest sequence, which is more than 20 nt long, is located after E4); *(iii*) their proximity to alternative exons; and *(iv)* the repeat lengths of certain imperfect repeats, such as those of the dinucleotides TA or GT in intron 0 (TA_356,_ GT_187_) (Fig. 1). A 58-nt motif (SSR_58_) that is repeated twice is located immediately upstream (precisely 10 nt) of the alternatively spliced E10 [73, 74]. Interestingly, the copy number of this long repeat seems to influence the splicing pattern of this exon [73]. Although no functional role has been demonstrated, notably, a dinucleotide GT repeat (x22: GT_22_) has been identified immediately downstream of E0 (precisely 362 nt from E0) in the human gene, as first reported by Andreadis *et al.* [75]. Compared with the mouse sequence, half of this human TG repeat corresponds with an insertion in a sequence that is well-conserved (76 % homology) between human and mouse E0. However, a similar 23-repeat TG sequence also exists in intron 0 of mouse *MAPT*, but it is located 7301 nt downstream instead of 362 nt downstream of E0 in a non-homologous region of the human gene. We also observed 4 different short regions containing CATC or CCAT repeats located immediately downstream (from 46 to 1200 nt) of the internal exon E1A, between E1 and E2 in the only *MAPT* transcript that is potentially subjected to nonsense-mediated decay (NMD) (*MAPT*-011 ENST00000571311) (Fig. 1, Fig. 4b).

### CpG islands and methylation

#### CpG islands

**Fig. 2:**
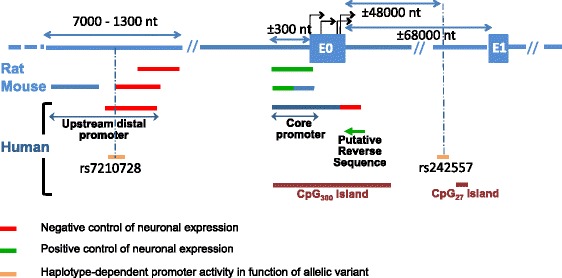
Organization of the rat, mouse and human Tau promoters. The lengths of the various regions of the promoter are not representative of their precise localizations but are dependent on the cloning technique. Thus, differences in the length of a determined region among species are not significant. Note the influence of allelic variants on promoter activity. CpG islands present in the promoter are indicated

As determined by CpG Islands Track Settings (UCSC), one long CpG island spans approximately 300 nt upstream to 3000 nt downstream of E0 in human *MAPT* (the total [G + C] increases to 75 % around the transcription start site, with as high as 10 % CpG dimers) [76–78]. This CpG island (CpG_300_) is located in the promoter region (Fig. 1, Fig. 2). Two additional but more limited sequences are present, including one containing 27 CpGs (CpG_27_) that is located 13 kb upstream of E1 and another containing 21 CpGs (CpG_21_) that is located immediately upstream of E4A (UCSC Genome Browser [79]). The CpG_21_ island is situated at the beginning of the short transcript MAP-010/ENST00000576238 (Fig. 1, Fig. 4b). Thus, we cannot ignore the possibility that this CpG island may be involved in the transcriptional regulation of this minor transcript (see section 3.1.3). However, no histone H3 lysine 4 trimethylation, another feature of promoter regions, was found in this third CpG island (CpG_21_) using CpG Islands Track Settings (UCSC genome browser) [76, 80]. In addition to these three CpG islands, other CpG islands can be identified using less restrictive conditions. For example, one CpG island was identified in exon E9 (198 nt/266) using MethPrimer (http://www.urogene.org/methprimer) [75, 81]. This type of CpG island is a 3′ island as defined by Gardiner-Garden and Frommer [76]; however, no methylation studies of this type of island have been reported. Another CpG island has been predicted in the coding region of the last exon, E13, using the same MethPrimer tool [81].

**Fig. 3:**
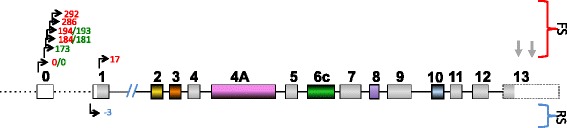
Transcription initiation sites (black perpendicular arrow) and termination sites (simple gray arrow). These sites enable the expression of the complete Tau transcript/protein. For other sites, see Fig. 4b. Red numbers: initiation sites according to the database. Green numbers: initiation sites described in [77]. FS: forward strand, RS: reverse strand

#### DNA methylation

Effective DNA methylation has been identified in both CpG_300_ and CpG_27_ islands; however, functional studies have focused primarily on the CpG_300_ island located around E0. In the brain, *MAPT* methylation varies according to the area analyzed [82, 83]. Interestingly, DNA hypomethylation has been observed in various pathologies, such as PSP, AD and PD [82, 83]. These differences have been observed in neuronal and non-neuronal cells in cases of sporadic AD [83]. *MAPT* hypermethylation leads to decreased Tau expression, suggesting that the hypomethylation observed in AD is associated with an increase in Tau protein expression and thus could participate in aggregation seeding and pathological propagation through the brain via a prion-like mechanism [83]. Concerning PD, hypermethylation in the cerebellum (the region spared in PD) and hypomethylation in the putamen (the region with the highest level of PD pathology) are consistent with reports on AD, with a possible neuroprotective effect of DNA hypermethylation by reducing *MAPT* expression. Furthermore, the neuroprotective effect of *MAPT* hypermethylation is somewhat illustrated by the fact that higher methylation in Parkinson leukocytes is associated with late-onset PD [82]. However, some contradictory results have been reported, including no changes in methylation in AD, PD and some cases of frontotemporal dementia (FTD) [84]. These differences might have resulted from the analysis method used (microarray analysis/pyrosequencing) and/or the brain region analyzed (hippocampus, frontal cortex/cerebellum, parietal cortex and temporal cortex).

### Haplotypes

#### Tau haplotypes and neurodegenerative disease development

More than 120 nucleotide changes across the *MAPT* gene have now been described, 60 % of which are primarily localized to two clusters in introns 9 and 10 [85]. An atypical pattern of linkage disequilibrium results in 2 major haplotypes: H1 and H2. Among the genetic variations, the H2 haplotype differs from H1 by the deletion of a 238 bp sequence upstream of Tau exon 10 (reviewed in [60]). Another feature of the H2 haplotype is the inversion of a 900 kb sequence, most likely due to non-allelic homologous recombination between the long coding repeats (LCRs) that surround this region [86, 87]. The frequency of H2 inversion differs between ethnic groups; the H2 haplotype is found primarily in Europeans and southwest Asians. Notably, the presence of various homologous LCRs in this short genomic region surrounding the *MAPT* locus could trigger other genomic rearrangements, leading to microdeletion/microduplication/microtriplication of this short genomic region [29–32]. H2 inversion seems to be a risk factor for genomic rearrangements such as microdeletions [88].

The H1 haplotype is relatively heterogeneous compared with H2. A total of 24 SNPs in the *MAPT* gene vary in the H1 background haplotype and enable classification of the gene into H1 sub-variants [89]. H1 haplotypes or sub-haplotypes are associated with increased risks of developing certain neurodegenerative diseases. Although first established for PSP and CBD, the list of pathologies is increasing and includes the following: neurofibrillary tangle-predominant dementia, PD, AD, primary progressive aphasia, and argyrophilic grain disease [72, 89–97]. However, Sobrido et al. have reported no association between the H1 haplotype and FTD [98] and a slight, but not significant, increase in the H2 haplotype frequency has been reported in Pick disease [99].

Notably, DNA methylation is increased in the H1 haplotype compared with that in the H2 haplotype and can be observed in both the blood and brain [82, 100]. Furthermore, differential methylation (hypomethylation or hypermethylation, depending on the analyzed site) could play a role in the association of the H1 haplotype with the development of PSP, particularly for the methylation sites within the 17q21.31 region where the *MAPT* gene is located [100].

#### Functional effects of the H1 haplotype on pathology

The functional effects of the H1 haplotype on the development of neurodegenerative diseases have been investigated at different levels.

PSP and CBD, the first pathologies associated with the H1 haplotype, display particular patterns of Tau isoform expression. Indeed, Tau isoforms containing sequences encoded by the alternative exon 10 of Tau mRNA are preferentially found in degenerating neurons, suggestive of altered Tau splicing. Consistent with these observations, the H1C sub-haplotype found predominantly in these pathologies seems to favor exon 10 inclusion compared with the H2 haplotype [101]. Caffrey *et al.* have also reported an increase in exon 3 inclusion for the H2 haplotype [102]. Further, these authors have suggested that the E3-encoded sequence could have a protective effect against these pathologies, consistent with the observed decrease in the fibrillar extension of Tau proteins when the E3-encoded sequence is present [103].

Using luciferase assays, the H1 haplotype promoter has been shown to be more efficient at promoting Tau transcription compared with the H2 promoter [104–106]; however, these data are somewhat controversial because allele-specific expression experiments have not detected a difference in expression between these two haplotypes [104–106]. Notably, using this method, the authors have shown a decrease in H1 haplotype expression during aging.

Some polymorphisms have been demonstrated to interfere with *MAPT* expression. Two allelic variants located upstream of E0 (rs7210728) (±3000 nt) and downstream of E0 (rs242557) (±48000 nt) have been identified as modulators of promoter activity according to haplotype (H1 versus H2) [101, 106] (Fig. 2). Notably, the second polymorphism, rs242557, is located only approximately 6800 nt upstream of the second CpG_27_ island (Fig. 1, Fig. 2).

The relationship between *MAPT* haplotype and Tau and/or α-synuclein pathologies has also been investigated by studying brains affected by AD or α-synucleinopathies, including PD and dementia with Lewy Bodies (LBs). Surprisingly, the H1 haplotype may be protective against Tau aggregation but favors α-synuclein aggregation in LBs [107–109]. However, the results may be dependent on the studied brain area, the pathology type, the aggregate type and other factors associated with these pathologies, such as ApoE/α-synuclein haplotype and amyloid aggregation. Thus, additional studies are needed to determine whether the *MAPT* haplotype has an effect on Tau or α-synuclein deposition. Another method used to ascertain the potential relationship between Tau haplotype and tauopathy development involves examining the possible relationships between *MAPT* haplotypes and biological markers. A diagnostic test first developed for AD based on three major biomarkers (amyloid peptide Aβ1-42, total Tau (t-Tau) and Tau phosphorylated at Thr181 (p-Tau)) of AD found in CSF has been proposed [110]. Unfortunately, conflicting data have been obtained for the H1C sub-haplotype regarding Tau levels in CSF [111, 112]. These two groups have also reported correlations between specific *MAPT* SNPs and Tau levels in CSF. Among these SNPs, at least some may be associated with an earlier stage at onset [111]. More recently, an N-terminal fragment of Tau has been identified in CSF as a new diagnostic marker for various neurodegenerative diseases associated with cognitive impairment [113]. If this result is confirmed, then investigating the relationship between Tau haplotypes and this new marker would be of interest.

## Regulation of Tau RNA expression and splicing

Tau mRNA expression was initially identified in the brain and, more precisely, in neurons by both northern blot and *in situ* hybridization analyses; it has since been detected in various tissues, including the cerebellum, kidney, muscle and testis, using more sensitive methods such as RT-PCR, microarray and RNA-Seq [57, 114–116]. However, although Tau protein expression in peripheral tissues has been clearly observed in rats [116], very little data concerning such expression in human tissues have been reported [117], with the exception of reports of Tau protein expression in pathological peripheral tissues [37, 41, 118].

Mature Tau transcripts contain up to 16 exons [3, 57]. The different exons have been identified and numbered, corresponding to their locations within RNA sequences from different animal, primarily human, bovine and murine, models. The first exon is generally named exon 0 (E0) in the literature, and exon 4A corresponds to the alternative exon located between exons 4 and 5. The exon numbers provided in the databases must be carefully considered when determining the functions of analyzed transcripts because different exons may be assigned the same number depending on the transcript analyzed.

Three Tau transcripts of 2 kb, 6 kb, and 9 kb in length are encoded by the *MAPT* gene (reviewed in [57]). These transcripts differ from one another by their splicing patterns, as demonstrated by the differences between the 6 and 9 kb transcripts [119], and their polyadenylation sites (2 kb versus 6 kb) [120]. The abundance of data in databases largely confirms the observed variations in splicing patterns and polyadenylation sites but also suggests the existence of several promoters, as detailed below.

### 5′ UTR/*MAPT* promoter

#### *MAPT* promoter identification in the E0 region

The *MAPT* promoter has been studied primarily in three different models, namely human, rat and mouse models. The *MAPT* promoter is characterized by a high G + C content as well as by the absence of TATA and CAAT boxes [65, 77, 121]. Several regions have been determined to play important roles in Tau transcription (Fig. 2).

First, the “core” promoter is located immediately upstream of the first exon (exon E0, previously named exon −1) (Fig. 2) [64, 65, 77, 101, 121]. More detailed studies of rat and mouse models have enabled differentiation between the two sub-sequences in this core region [121, 122] (Fig. 1). The sequence located 5′ of the core sequence is involved in neuronal-specific expression in rats and mice but not in humans, whereas expression of the 3′ sequence is not tissue dependent [77, 121–123]. However, this apparent difference in the neuronal specificity of the core promoter between mice and humans may be due to differences in the cell type and the length of the promoter sequence used in constructs, as suggested by Maloney and Lahiri [64]. These authors have reported the influence of E0 on human promoter activity and have identified a neuronal-specific sequence that includes the last 10 nt of E0 and the first 200 nt of the intronic sequence located immediately downstream that negatively regulates promoter activity (Fig. 2).

Other distal sequences (approximately −7000 to −1500 nt upstream of exon E0) are involved in promoter activity [64, 101, 121] (Fig. 2). The 3′ end of this sequence (approximately −3000 to 1500 nt) negatively regulates Tau expression in rats and humans [64–66, 101, 121]. The 5′ end of this sequence (approximately −7000 to 3000 nt), which has only been studied in mice, is also involved in promoter activity [121]. In addition, a putative reverse sequence located immediately downstream of E0 might be involved in human *MAPT* regulation (Fig. 2) [64]. However, these authors could not exclude interference from another promoter because of identification of a hypothetical gene, LOC100128977, immediately upstream of and in the opposite orientation as the *MAPT* gene [124].

**Fig. 4:**
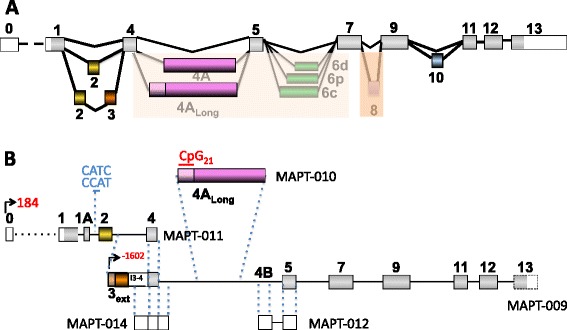
Splicing patterns of human Tau transcripts. **a** Classical splicing pattern described in the literature. The insertion of exons 4A and 6 rarely occurs in the brain (shaded in pale orange) and varies according to which 3’ splice site is used. E8 insertion (shaded in dark orange) has not been reported in humans but has been described in different animal models and in the human Ensembl database. **b** Hypothetical splicing according to the Ensembl database. The *MAPT* numbers correspond to those in the Ensembl database. White, non-coding regions; gray, constitutive coding exons. Yellow, orange, pink, green, purple and blue represent the alternative exons 2, 3, 4A, 6, 8 and 10, respectively

According to the technique used, several initiation sites for Tau transcription have been reported in both rats and humans [65, 77]. The human transcript sequences published in databases confirm the diversity of the transcription start sites. However, some discrepancies exist between the sites described in the literature and those in databases. In particular, the initiation sites of the published sequences are located in E0, whereas the Ensembl database shows that various initiation sites are present all along the gene in addition to those in E0 (Fig. 3, Fig. 4b) [125]. Three of these identified sites are common between the literature and databases.

#### *Trans* factors

As demonstrated by electrophoretic mobility shift assay (EMSA), the transcription factors SP1 and AP2 bind to the Tau core promoter region and are necessary for promoter activity [121, 122]. Another DNA sequence located between the SP1 and AP2 sites influences promoter activity; however, the transcription factor involved remains to be identified [122]. EMSA has also revealed that SP1, progesterone receptor (PR) and retinoic acid receptor (RAR) bind to the human exon E0 sequence, although their functionalities remain to be demonstrated [64].

The human *MAPT* promoter region also contains putative binding sites for various other transcription factors, such as Nrf1, MTF1, MBF1, MepI, and GCF [64, 101]. Some of these sites are shared between the H1 and H2 haplotypes, such as the AP2 and SP1 sites mentioned above. Interestingly, among these common sites, four sites corresponding to an Aβ-interacting domain have also been identified in the distal region of the Tau promoter [64, 126]. Aβ is a pathological peptide generated via deregulated proteolysis of amyloid precursor protein (APP). This peptide aggregates during AD and leads to the observed pathology of this disease according to the amyloid cascade hypothesis. This aggregation/accumulation of Aβ is not a feature common to all tauopathies, but it does occur in some, such as AD and Down syndrome. Several studies have suggested that the pathological effect of Aβ is mediated by Tau (reviewed in [127, 128]). Thus, determining whether the Aβ-interacting domain actually regulates Tau expression directly and thus participates in the development of Tau pathologies in which both amyloid and Tau aggregates are present would be of interest.

#### Does *MAPT* have only one promoter?

Alternative initiation transcripts largely contribute to transcriptome and proteome diversity, and more than 40 % of genes have at least two promoters, as demonstrated in flies (reviewed in [129, 130]). Although the region around E0 contains the only promoter for *MAPT* that has been described in the literature, Andreadis [57] hypothesized the existence of additional Tau promoters to explain variations observed in neuronal specificity and NGF susceptibility of Tau expression according to the different Tau transcripts [77, 119, 131]. Currently, new data are available in databases that reinforce this hypothesis; at least 6 other exons distal to E0 have been identified as the first exons of various transcripts.

One of the potential additional promoters may be associated with the initiation of transcription at exon E1. Indeed, considering the number of identified transcripts, E1 is the most frequent exon where transcription is initiated after E0 (transcripts initiated at E0: 9 and E1: 5) for the H1 haplotype, further suggesting the possible existence of an unidentified promoter. E1 is located 68000 nt distal to E0. Similar to E0 [57], sequence analysis has revealed no TATA or CAAT consensus binding sites in E1; however, a GC box specific for SP family members is present near this exon [132]. Indeed, one potential SP1 site and five sites for AP-2α binding have been identified 200 nt upstream of E1 using the ALGGEN server [133]. The absence of E0 would result in a change in the 5′ UTR length but would not alter the coding sequence because E0 is a non-coding exon, while E1 is a partially coding exon (Fig. 1). On the reverse strand (H2 haplotype), transcripts listed in the Ensembl database begin primarily with E1 [125] (Fig. 3).

The other initiating exons near the E2, E3, E4A and E5 regions are located more than 10,000 nt and up to 25,000 nt distal to E1, suggesting that more than one alternative promoter may exist. Interestingly, the CpG_21_ island is located immediately upstream of E4A at the beginning of the short transcript MAP-010/ENST00000576238, as described above in the CpG island section (Fig. 4b).

### Tau splicing

Here, we will not describe the splicing mechanisms in detail because they have been discussed in previous reviews; however, we will focus on the Tau splicing pattern because splicing is an indirect mechanism of protein function modulation via sequence modification [24, 57, 61, 134].

#### Published data

Tau alternative splicing is dependent on the developmental stage, tissue and species.

##### Central nervous system (CNS)

The following are generally recognized facts regarding Tau splicing in the human CNS.

(i) In the fetal brain, only one isoform is predominantly expressed, with insertion of the constitutive coding exons (E1, E4, E5, E7, E9, E11, E12, and E13/14). E1 is considered a coding exon because it contains the initiating ATG codon. In addition, there are no reports in the literature suggesting the presence of non-coding E0 during the fetal stage [3, 57, 135, 136].
(ii) E2, E3 and E10 are specific to the adult brain (Fig. 4a). Consistent with this statement, 6 protein isoforms have been identified in the human adult brain that contain sequences encoded by exon 2, exon 3 and/or exon 10. Generally, these 6 isoforms are named 2N3R, 1N3R, 0N3R, 2N4R, 1N4R, and 0N4R. The designations 0 N, 1 N and 2 N indicate the exclusion of E2 and E3, the inclusion of E2 and the inclusion of both E2 and E3, respectively. Notably, the insertion of E3 is dependent on the presence of E2, which corresponds with an atypical feature of the splicing event. Globally, E3 inclusion can be considered a rare event compared with E2 insertion or complete Tau transcription, as evidenced by RT-PCR [114, 137]. Conversely, 3R and 4R indicate the exclusion and inclusion of E10 [115, 138], respectively, which has been previously reviewed [3, 139].

##### Peripheral tissues

Considering peripheral tissues, including the peripheral nervous system (PNS), the alternative exons described above are often included with other exons that are nearly absent in the CNS. Indeed, exon 4A (E4A) is present specifically in the PNS and retina, and exon 6 (E6) is found in the spinal cord and skeletal muscle. Interestingly, the lengths of these latter two exons, E4A and E6, are modulated by the choice of 3′ splice site. Indeed, two possible 3′ splice sites exist for E4A (E4A, E4A_Long_), and there are 3 splice sites for E6 (E6c, E6p, E6d) [41, 117, 137] (Fig. 4a). However, although isoforms containing E4A_Long_, E6p, and/or E6d have been reported in the literature, they are not present in human databases (Ensembl, UCSC). These observations bring into question the frequencies at which these different 3′ splice sites are used. Considering the possible co-insertion of alternative exons, in ENSEMBL/UCSC sequences, E4A is always inserted when E6 or E6 and E8 are included with E2, E3 and E10. However, an E4A-containing isoform without E6 but containing E2, E3 and E10 has been cloned [140]. Furthermore, in Ensembl database, the isoforms containing exon 6c also contain exons 2, 3, 4A and 10 with or without exon 8, although studies by Andreadis’s group have demonstrated that all combinations of E6c, E6p, and E6d with the other alternative exons are possible [141]. The inclusion of E6p and E6d results in the translation of a truncated protein that lacks microtubule-binding sites because of a change in the reading frame that introduces a stop codon. Tau proteins containing E6c- or E6d-encoded sequences have been identified in several tissues, including brain and muscle tissues, using specific antibodies [142, 143]. These new forms, although minor, could interfere with the role of Tau in axonal transport [144, 145]. Note also the rare but possible inclusion of E6 in the fetal brain [57].

#### Suggestions from databases

Recent data from databases suggest that the splicing pattern of *MAPT* may be more complicated than suggested by the primary characteristics described above, although particular splicing events are most likely rare. Although the majority of transcripts described in databases are coding transcripts, some are not (Fig. 4b). Non-coding transcripts have been identified that correspond primarily to isoforms with the deletion of at least one constitutive exon. One such non-coding transcript (*MAPT*-011 ENST00000571311) (Fig. 4b) may be processed by NMD. Notably, none of these variants have been described in the literature, although they are listed in Ensembl. Their existence and potential roles (perhaps as lncRNAs) remain to be investigated and confirmed.

##### Splicing and H1/H2 haplotypes

A comparison of the transcripts from the forward (H1) and reverse (H2) strands that have been published in databases such as Ensembl has revealed that the haplotype might affect some splicing events. This situation has already been reported for E10 (see the sub-chapter “Functional consequence of H1 haplotype on pathology”).

##### Alternative 5′ UTR/alternative first exon

In the literature, E0 is always described as the first exon in all *MAPT* transcripts. However, when analyzing different *MAPT* transcripts in databases such as Ensembl, it is apparent that E0 can be present or absent in the H1 haplotype; thus, it may be considered an alternative exon due to alternative promoter usage. Furthermore, E0 inclusion is less frequent in the sequences of transcripts from the reverse strand (H2 haplotype) (Fig. 3). Several possible initiating transcription sites may exist in E0 as described above (see the sub-chapter regarding promoters), leading to different possible lengths of E0 and of the 5′ UTR because E0 is a non-coding exon (Fig. 3). According to the Ensembl database, the length of E1 may also vary depending on the first exon that is included (E0/E1). Indeed, E1, a partially coding exon, is 150 or 133 nt long when initiated from E0 or E1, respectively, on the forward strand. For H2 (the reverse strand), in the absence of E0, the length of E1 is three nucleotides longer at its 5′ side (153 nt) or 17 nucleotides shorter at the ATG codon (133 nt). These variations in E1 length do not affect the sequence of the translated protein because the 5′ end is non-coding; therefore, this length variation only results in modification of the 5′ UTR of the transcript (Fig. 2).

##### Additional exons

The possible insertion of additional exons, although not yet identified in the published human transcripts, is suggested by data in the Ensembl database. This possibility is the case for E8, which was first identified in bovines and then in certain species such as rhesus monkeys [146, 147] but has not been described in humans [148] (Fig. 4a). However, according to the Ensembl database, transcripts containing E8 include all of the alternative exons except for E0, which can be included or excluded (*MAPT*-203 ENST00000344290, *MAPT*-004 ENST00000415613). Studies of different animal species have shown that E8 insertion is possible in the absence of certain alternative exons. In particular, 4 transcripts containing E8 have been identified in cows [148]. All of these transcripts also contain E2 and E10 but lack E4A. These transcripts differ from each other by the presence or absence of E3 or E14. In mice, only one transcript (*MAPT*-006/ENSMUST00000106993) has been identified with E8, and this transcript contains E10 as the only other alternatively spliced exon.

New exons that have never been reported in the literature are present in certain transcripts. The insertion of most of these new exons results in truncated transcripts at the 5′ and/or 3′ ends of Tau mRNA (Fig. 4b). For example, the exon located between E1 and E2, named E1A, is present in the only transcript that potentially undergoes NMD *(MAPT*-011, ENST00000571311/ENST00000625688) (Fig. 4b)*.* In addition, we identified exon E4B, which is positioned between E4A and E5 and is spliced to E5, producing a non-coding but processed transcript composed of two exons (*MAPT*-012, ENST00000577017/ENST00000627800) (Fig. 4b).

Other exons generated by the extension of known exons have been found in particular isoforms in Ensembl database. Exon E4A_Long_ has been described in *MAPT* transcripts as another spliced form of exon 4A [41] (see above). E4A_Long_ may also be the only exon in a short transcript listed in Ensembl database (*MAPT*-010 ENST00000576238/ENST00000625417) (Fig. 4b). Another example is extended E3 (E3_Ext_, 5658 nt versus E3, 87 nt), which may be the first exon in the *MAPT*-009 transcript (ENST00000576518/ENST00000626880) (Fig. 4b). Exon E3_Ext_ is composed of exons E3 and E4, the 1603 nt upstream of E3 and the entire retained intron between E3 and E4 (3903 nt) (Fig. 4b). E3_Ext_ may be a partially coding exon because an ATG is located 100 nt before E4 and is spliced to exon 5, encoding a protein that lacks part of the 5′ region that contains the acidic projection domain (Fig. 4b). Because the alternative exons E4A, E6, and E10 are excluded from this transcript, it may be translated more precisely in this shortened form during fetal development. Interestingly, if this transcript exists, then it has a short 3′ UTR. Another example of an extended exon is extended exon E4 (E4_Ext_), which corresponds to exon E4 in-frame with partial intronic sequences (2883 nt upstream and 2066 nt downstream) and which may be the only exon in the non-coding transcript *MAPT*-014 (ENST00000572440/ENST00000628437) (Fig. 4b). Certainly, the existence of these two latter transcripts must be confirmed under physiological conditions.

#### Splicing and pathology

Defects in splicing can be pathological events that lead to or have roles in tauopathy-type neurodegenerative processes. Indeed, missplicing has been clearly associated with certain familial tauopathies, such as FTDP-17 and DM (reviewed in [24, 134]). These pathologies have been associated with either mutations in *MAPT cis*-elements (FTDP-17) (review in [61]) or with microsatellite expansion in the non-coding regions of various genes, leading to variations in the expression of splicing factors belonging to the CELF and MBNL families (DM, reviewed in [24])*.* Notably, numerous mutations in *MAPT* are located in or near E10 (upstream and downstream), favoring E10 inclusion either by destabilizing a stem–loop structure that encourages access to the 5′ splice site or by modifying splicing factor or U1/E6 snRNP binding sites (reviewed in [61]). For sporadic tauopathies, the *MAPT* haplotype may influence splicing, as described above. Moderate Tau missplicing events have also been observed in numerous other tauopathies, such as AD (reviewed in [24]). In AD and PSP, modified miRNA expression may result in alterations in the expression of certain splicing regulatory factors, which would then influence Tau splicing [149, 150]. As such, the splicing pattern of *MAPT* transcripts could be determinant in tauopathy-type neurodegenerative processes. Consistent with this hypothesis, none of the mouse transgenic models have reproduced Tau pathology in the absence of a Tau mutation in the coding region. However, Tau pathology has been observed in mouse models when the human Tau gene is expressed in the absence of endogenous *MAPT* expression. These observations suggest that the correct ratio between the differentially expressed isoforms is determinant in the tauopathy process in the absence of Tau coding mutations. Thus, the difficulty in obtaining animal models that reproduce tauopathies in the absence of a Tau mutation most likely results from the *MAPT* splicing pattern, which differs among animal species [151–155]. For example, exon 10 inclusion is highly increased in mouse and rat brains, whereas exon 2 inclusion is observed less frequently in mice compared with humans. Similarly, differences in the 3′ ends of the transcripts, as described below (section 3.3), may stem from splicing differences that potentially interfere with the human specificity of the tauopathy process. However, the contribution of other undetermined factors cannot be excluded at this point.

### 3′ UTR/alternative termination sites: Roles of RNA stability and localization

The 2 and 6 kb forms of mature Tau RNA are generated from different 3′ UTRs. Indeed, two alternative polyadenylation sites followed by a short poly(A) sequence located at a distance of approximately 4000 nt have been found in different animal species [74, 115, 120, 156] (Fig. 3). Thus, at least two transcription termination sites could be used, resulting in transcripts with short or long 3′ UTRs. Interestingly, the first polyadenylation site is located within an intronic sequence that is deleted in some transcripts from various animal species. Indeed, in some Ensemble database transcripts from mouse, rat and cow, E14 is separated from E13 by a short intronic sequence of approximately 900 nt [148, 157]. The splicing of E13 to E14 results in extension of the C-terminus of the protein and truncation of the 3′ UTR. In humans, the short intronic region between E13 and E14 is always retained, as described in the literature [74] and databases such as Ensembl. Thus, the splicing of E13/E14 may be a feature that differs between animals and humans.

The 3′ UTR may contain *cis*-elements that decrease Tau RNA expression. Indeed, binding sites for miR-34a and miR-34c-5p have been reported that lead to decreased Tau RNA and protein expression [45, 156]. Interestingly, increased Tau expression and decreased miR-34C-5p expression have been correlated with the chemoresistance of gastric cancer to paclitaxel [45]. Recent studies have demonstrated a role of miR-485-5p in axonal development and Tau expression downregulation during synaptic plasticity in hippocampal rat neurons. The Tau 3′ UTR sequence has two possible miR-485-5p binding sites [158] and contains a target sequence for miR-132-3p, which is strongly downregulated in the brains of AD patients [149]. However, a mutation in the binding site does not completely abolish miR-132 activity, suggesting that other indirect mechanisms exist by which miR-132 affects Tau expression.

In rat transcripts, a 240-nt region in the 3′ UTR has been implicated in RNA stability and axonal localization [159–161]. Several proteins, such as HuD (an Elav protein family member), insulin-like growth factor mRNA-binding protein IMP-1, Ras-regulatory protein G3BP, interleukin enhancer binding factor 3 (Ilf3) and NF90, bind to this sequence and are potentially involved in the axonal localization of these transcripts [162]. Unfortunately, no such data are available in humans, and these data must be generated to determine whether similar mechanisms protect human RNA and guide transcripts to their proper locations.

## Conclusion: The hidden facets of Tau gene expression

The aim of this review was to highlight the different steps of *MAPT* gene expression regulation at the DNA and RNA levels. We focused on the following three aspects of the influence of DNA sequence on *MAPT* expression: repeated sequences, CpG islands and haplotypes. Notably, scarce data concerning repeated sequences have been reported in the literature, whereas CpG islands and haplotypes are beginning to be well documented. Functional interest in repeated sequences has recently been described in the literature for other genes. For *MAPT*, the report of the role of SSR_58_ in E10 insertion confirms the interest in such studies [73]. Concerning the regulation of RNA expression, we focused on the potential roles of UTR sequences and alternatively spliced isoforms. Additional data are necessary to confirm or exclude the possible existence of another promoter. Furthermore, differences exist between isoforms reported in the literature and those reported in databases, suggesting that some splicing possibilities remain to be explored. Data regarding Tau isoforms reported in the literature primarily concern the nervous system. However, Tau (transcripts/proteins) is expressed in other tissues, and its expression may play roles in some pathologies, such as cancer and IBM. Thus, increasing our understanding of the isoforms expressed in these different tissues and of the regulation of their expression, which may differ from those in the brain (for example, different promoters and polyadenylation sites) is of interest. In addition to the isoforms described in the literature, the data from databases suggest the possible existence of shortened Tau isoforms, non-coding transcripts and one transcript that may undergo NMD, thereby bringing into question the roles of these hypothetical forms. Finally, the Tau sequences present in databases also suggest the possible existence of a long non-coding *MAPT* RNA and 3 *MAPT* anti-sense isoforms; however, these sequences have not yet been reported in the literature. Therefore, they were not discussed here because of the lack of information. Knowledge of these particular Tau transcripts will most likely increase our understanding of Tau expression regulation.
